# Porcine epidemic diarrhea virus manipulates IMPDH-dependent nucleotide biosynthesis to facilitate replication

**DOI:** 10.1128/jvi.01736-25

**Published:** 2026-01-09

**Authors:** Shuting Zhou, Houde Zhao, Junrui Zhu, Yanjun Zhou, Zhibiao Yang, Zhe Wang

**Affiliations:** 1 Shanghai Collaborative Innovation Center of Agri-Seeds / School of Agriculture and Biology, Shanghai Jiao Tong University 117750, Shanghai, China; 2 Shanghai Veterinary Research Institute, Chinese Academy of Agricultural Sciences 118161 , Shanghai, China; 3 Shanghai Key Laboratory of Veterinary Biotechnology, School of Agriculture and Biology, Shanghai Jiao Tong University 117750, Shanghai, China; Loyola University Chicago - Health Sciences Campus, Maywood, Illinois, USA

**Keywords:** porcine epidemic diarrhea virus (PEDV), IMPDH, merimepodib, nucleotide biosynthesis

## Abstract

Porcine epidemic diarrhea virus (PEDV) causes acute intestinal disease in pigs and remains a major threat to the global swine industry due to its high morbidity and mortality in neonatal piglets. To investigate host metabolic alterations upon PEDV infection, we performed untargeted metabolomic profiling in LLC-PK1 and Vero E6 cells. Pathway enrichment analysis revealed significant changes in nucleotide metabolism, cofactor biosynthesis, amino acid biosynthesis, and purine metabolism. Notably, PEDV infection led to divergent regulation of purine metabolism in the two cell types—upregulation in Vero E6 cells and downregulation in LLC-PK1 cells at 18 h post-infection. We further identified inosine monophosphate dehydrogenase (IMPDH), the rate-limiting enzyme in guanine nucleotide biosynthesis, as a critical host factor for PEDV replication. Both genetic knockdown of IMPDH2 and pharmacological inhibition using merimepodib (VX-497, MMPD) significantly reduced viral RNA levels and impaired replication. These treatments also suppressed host nucleotide biosynthetic activity. Together, our findings demonstrate that PEDV hijacks the IMPDH-dependent guanosine biosynthesis pathway to support its replication and identify IMPDH as a promising host-directed antiviral target against PEDV.

**IMPORTANCE:**

PEDV poses a major global threat to swine health. This study uncovers a key mechanism of pathogenesis: PEDV exploits host nucleotide metabolism, inducing significant reprogramming with emphasis on purine biosynthesis. Comparative infection of porcine (LLC-PK1) and primate (Vero E6) cells revealed cell-specific metabolic adaptations. Crucially, we identify inosine monophosphate dehydrogenase (IMPDH), the rate-limiting enzyme for guanosine biosynthesis, as an essential host dependency factor for PEDV replication. Inhibiting IMPDH genetically or pharmacologically significantly reduced viral titers, validating it as a critical vulnerability. These findings reveal a novel mechanism by which PEDV hijacks host metabolism and establishes IMPDH as a promising host-directed therapeutic target for combating this economically devastating virus.

## INTRODUCTION

Porcine epidemic diarrhea virus (PEDV) is an enteric pathogen classified in the genus *Alphacoronavirus* within the family Coronaviridae (1). It causes porcine epidemic diarrhea (PED), characterized by acute watery diarrhea, vomiting, and dehydration, with mortality rates exceeding 90% in neonatal piglets. Since its first identification in the United Kingdom in 1971 (2), PEDV has spread globally, and the emergence of highly virulent mutant strains in recent years has posed renewed challenges to swine health. In 2010, variant PEDV strains with increased pathogenicity and antigenic drift emerged, rendering existing vaccines and therapeutics less effective (3). Therefore, there is an urgent need to develop novel antiviral strategies against PEDV.

Viruses, as obligate intracellular parasites, depend on host metabolic pathways to generate energy and biosynthetic intermediates required for replication (4, 5). Many viruses, including positive-strand RNA viruses, have evolved mechanisms to reprogram host metabolism to meet their replicative needs (6–9). This includes modulation of metabolic enzymes and fluxes that support nucleotide, amino acid, and lipid biosynthesis. For example, picornaviruses rewire nucleotide metabolism to facilitate genome replication (10), SARS-CoV-2 exploits folate-mediated one-carbon metabolism (11), and Newcastle disease virus (NDV) enhances nucleotide biosynthesis to support viral proliferation (12). African swine fever virus (ASFV) also reshapes host energy and amino acid metabolism by increasing acylcarnitine levels (13, 14). However, despite its significance, only a few studies have addressed the metabolic changes during PEDV infection (15, 16).

Nucleotides are essential not only for cell proliferation but also for viral genome synthesis (17, 18). Consequently, the nucleotide biosynthesis pathway has become a key target for the development of antiviral and anticancer therapies (19). Inosine monophosphate dehydrogenase (IMPDH) catalyzes the conversion of inosine 5′-monophosphate (IMP) to xanthosine 5′-monophosphate (XMP), a rate-limiting step in the *de novo* synthesis of guanine nucleotides. Inhibition of IMPDH has been shown to suppress the replication of various viruses (20, 21). Merimepodib (VX-497, MMPD), an IMPDH inhibitor originally developed as an immunosuppressant, has demonstrated broad-spectrum antiviral activity against viruses, including HCV, Zika, Ebola, and FMDV (20, 22, 23). During the COVID-19 pandemic, MMPD was tested in combination with remdesivir in a phase 2 clinical trial (24). Its antiviral activity is attributed to the depletion of intracellular guanine nucleotide pools, which are essential for viral RNA synthesis.

In this study, we performed untargeted metabolomic profiling of PEDV-infected LLC-PK1 and Vero E6 cells to investigate how the virus modulates host metabolism during infection. We observed distinct alterations in purine metabolism between the two cell lines and identified IMPDH2 as a critical host factor for PEDV replication. Pharmacological inhibition of IMPDH with MMPD, as well as siRNA-mediated knockdown of IMPDH2, significantly suppressed nucleotide biosynthesis and viral replication *in vitro*. These results reveal a novel metabolic dependency in PEDV infection and highlight IMPDH as a promising target for host-directed antiviral therapy.

## MATERIALS AND METHODS

### Viruses, cell lines, and test compounds

PEDV strains *SHpd/2012*, HLJ, and AH2018 were maintained in our laboratory. Vero E6 cells and IPEC-J2 cells were cultured in Dulbecco’s modified Eagle’s medium (DMEM, Gibco), which was supplemented with 10% fetal bovine serum (FBS) (Viva Cell). LLC-PK1 cells were cultured in Minimum Essential Medium (MEM, Gibco, USA) supplemented with 10% FBS. All cell lines were preserved in our laboratory. Merimepodib was purchased from ApexBio (B1112).

### Cytotoxicity assays

The cell viability assay was performed using the Cell Counting Kit-8 (TOPSCIENCE, Shanghai, China). Vero E6, LLC-PK1, and IPEC-J2 cells were cultured in a 96-well plate with a series of MMPD concentrations. After 24 h of incubation, 10 μL of the reagent was added to each well. The absorbance at 450 nm was measured after 2 h of incubation of the cells with the reagent.

### Antiviral activity assay

Cells were exposed to the virus at 37°C for 1 h to facilitate viral attachment. Following this, the supernatant was discarded, and the cells were rinsed with PBS. Then, MMPD was added after viral infection at specified concentrations. After an additional 12 h of incubation, the cells and supernatant were harvested for inhibition assessment.

### Effect of MMPD on PEDV life cycle

For the virus attachment assay, LLC-PK1 and IPEC-J2 cells were separately pretreated with MMPD (30 μM) or DMSO for 1 h at 37°C. Following this, the cells were infected with PEDV (0.1 MOI) at 4°C for 15 and 60 min. Then, the cells were washed three times with ice-cold PBS. The PEDV genomic RNA levels were quantified using RT-qPCR, while the protein levels of PEDV N were detected by Western blot.

For the virus internalization assay, LLC-PK1 and IPEC-J2 cells were first infected separately with PEDV (0.1 MOI) at 4°C for 1 h. The cells were washed three times with PBS and then incubated with fresh maintenance medium containing MMPD (30 μM) or DMSO at 37°C for 1 h and 2 h. After washing three times with PBS, PEDV genomic RNA levels and N protein in different groups were analyzed using RT-qPCR and Western blot, respectively.

In the virus replication assay, LLC-PK1 and IPEC-J2 cells were inoculated with PEDV (0.1 MOI) at 37°C for 1 h. After washing three times with PBS, fresh maintenance medium was added. At 4 hpi, the medium was replaced with fresh DMEM containing either MMPD (30 μM) or DMSO. Cells and supernatant were collected at 6, 8, and 10 hpi. The levels of PEDV genomic RNA were measured using RT-qPCR, while the levels of PEDV N protein were analyzed by Western blot.

For the virus release assay, LLC-PK1 cells and IPEC-J2 cells were infected with PEDV (0.1 MOI) for 1 h. At 10 hpi, the cells were washed three times with PBS, and the medium was replaced with fresh DMEM containing MMPD (30 μM) or DMSO. Supernatants were collected at 10.5 hpi, 11 hpi, 12 hpi, and 14 hpi. The PEDV genomic RNA in the supernatants was analyzed using RT-qPCR, and PEDV N protein was assessed by Western blot. The supernatant was also analyzed for viral titer.

### Indirect immunofluorescence analysis

The cells were washed with PBS and fixed with 80% ice-cold ethanol for 45 min at 4°C. PEDV N monoclonal antibody (mAb) and goat anti-mouse IgG AF 488 were used as the primary and secondary antibodies, respectively. Nuclei were stained with 4,6-diamidino-2-phenylindole (DAPI), and fluorescence was observed using the Invitrogen EVOS FL Auto Cell Imaging System.

### Real-time PCR

RNA was extracted from the cells using the EZ-10 Total RNA Mini-Preps Kit (B618583, Sangon Biotech, China). RT-qPCR analysis was performed using the Hieff qPCR SYBR Green Master Mix (11,201ES03, YEASEN, China), according to the manufacturer’s instructions. The primers used in this study are listed in Table S1.

### Western blot analysis

Cells were lysed with RIPA Lysis Buffer (HY-K100, MCE, USA), separated by SDS-PAGE using the Color PAGE Gel Rapid Preparation Kit (10%; Epizyme), and transferred to polyvinylidene difluoride (PVDF) membranes. PEDV N monoclonal antibody (mAb), anti-IMPDH2 rabbit monoclonal antibody (mAb, T58727M, Abmart, China), and anti-β-actin mouse monoclonal antibody (mAb; A00702, Genscript, China) were used as primary antibodies. ECL Western blot substrate (SB-WB012, Share-Bio, China) was used to visualize the signals, which were then detected using the Tanon-5200 Multi-Infrared Imaging System.

### TCID_50_ assay

Vero E6 cells were seeded in a 96-well plate. The supernatant was removed, and cells were washed three times with PBS. Then, the cells were inoculated with serially diluted virus (10^−1^ to 10^−12^ dilution) at 37°C for 5 days. Cytopathic effects were observed daily, and the TCID_50_ value was calculated using the Reed-Muench method.

### RNA interference

RNA interference was performed by transfecting siRNA targeting pig IMPDH2 and a control siRNA. The siRNA sequences used are listed in Table S1. The siRNAs were transfected into LLC-PK1 and IPEC-J2 cells using Lipofectamine RNAiMAX (13778150, ThermoFisher, USA). The efficiency of RNA interference was evaluated by Western blot analysis.

### Exogenous guanosine supplementation analysis

LLC-PK1 cells were plated into 12-well plates in medium supplemented with 10% FBS and pretreated with guanine for 6 h. After infection with PEDV, the cells were incubated with MMPD (30 μM) and guanine for 12 h.

### Metabolite profiling

Virus-infected or mock-infected LLC-PK1 cells and Vero E6 cells were used for global metabolic profiling. LLC-PK1 cells and Vero E6 cells, treated with or without MMPD (30 μM) or in mock groups, were infected with PEDV (0.1 MOI) or maintained in culture medium for 12 h. Moreover, LLC-PK1 cells treated with siRNA targeting IMPDH2 or control siRNA for 36 h were then infected with PEDV for 12 hpi. Cell samples were washed with physiological saline and homogenized. Subsequently, the samples were incubated at −20°C for 1 h and then centrifuged for 10 min at 13,400 *× g* at 4°C. The supernatant was collected in a new Eppendorf tube for vacuum freeze-drying and resuspended in 1 mL of methanol/water (1:1, vol/vol) by vortexing. Finally, the samples were centrifuged for 10 min at 13,400 *× g* at 4°C, and the supernatant was transferred to autosampler vials for further analysis using the Thermo Scientific Orbitrap Exploris 120. Differential metabolites were selected (*P* < 0.05 and variable important in projection >1).

### Statistical analyses

The results are presented as means ± SD. Student’s *t*-test was used to compare the data between the treated and control groups. Statistical significance is indicated by asterisks (*P < 0.05; **P < 0.01; ***P < 0.001; ns = not significant). All statistical analyses and calculations were performed using GraphPad Prism 9.0.

## RESULTS

### Metabolic profiling of PEDV-infected cells

To investigate PEDV-induced metabolic reprogramming and compare the host metabolic responses between two permissive cell lines, LLC-PK1 and Vero E6, an untargeted metabolomics analysis was performed using ultra-high-performance liquid chromatography coupled with tandem mass spectrometry (UHPLC-MS/MS). Both PEDV-infected and mock-infected cells were harvested at various time points post-infection for comparative analysis.

**Fig 1:**
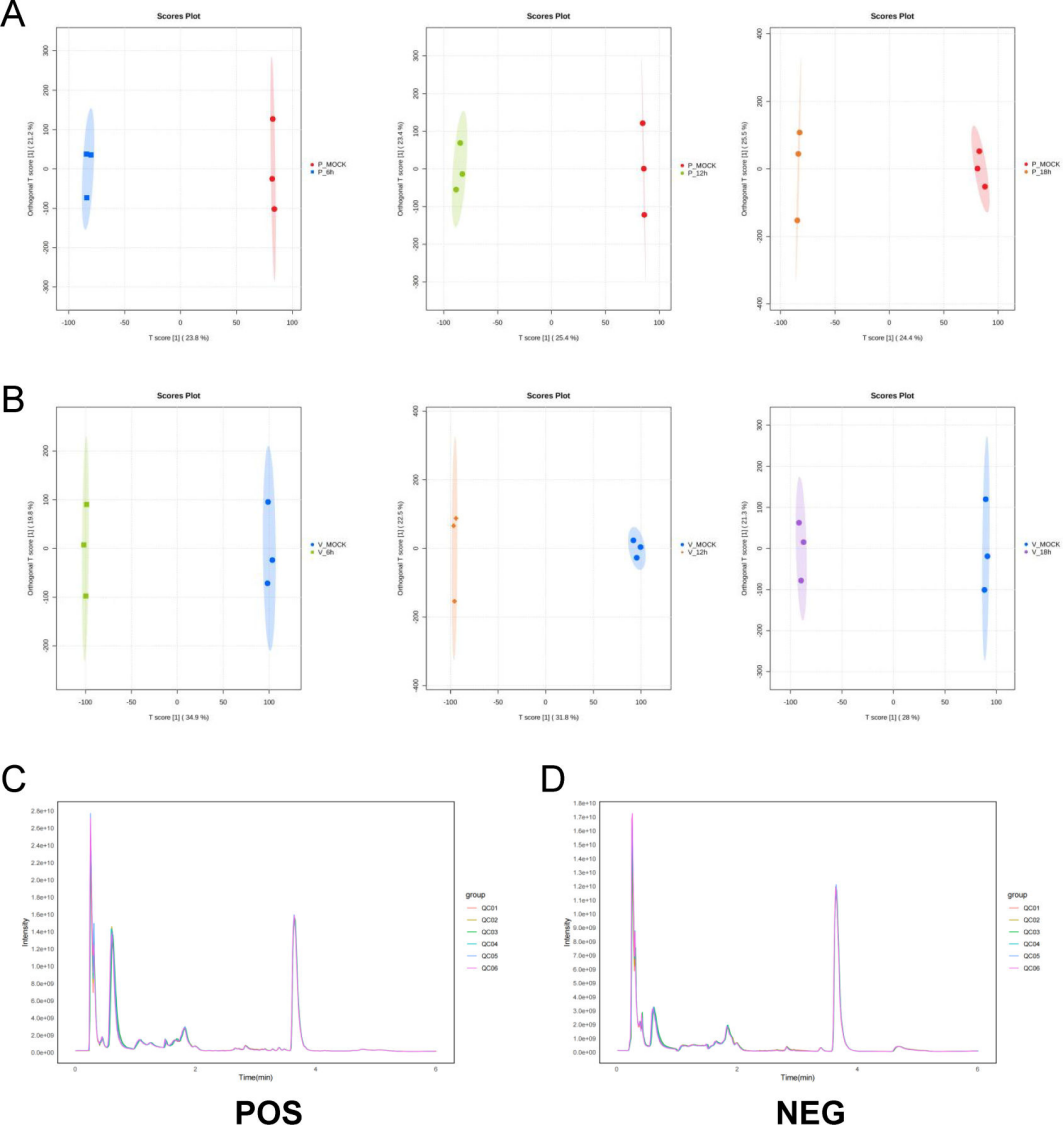
QC analysis of the mock‐infected and PEDV‐infected cells at the indicated time points. The OPLS‐DA model was obtained from the UHPLC-MS/MS metabolomic profiles of LLC-PK1 (P) samples (**A**) and Vero E6 (V) samples (**B**). (**C**) TIC overlap diagrams of untargeted metabolomics data in positive ion mode. (**D**) TIC overlap diagrams of untargeted metabolomics data in negative ion mode.

Orthogonal partial least squares discriminant analysis (OPLS-DA) was used to evaluate group separation and global metabolic differences between infected and control samples (Fig. 1A and B). Additionally, total ion current (TIC) chromatograms demonstrated high reproducibility across biological replicates, with consistent retention times and peak intensities observed in all groups (Fig. 1C and D), confirming the robustness and technical reliability of the metabolomics data.

These results establish a stable infection model and indicate that PEDV infection induces distinct alterations in the host metabolome in both LLC-PK1 and Vero E6 cells.

### PEDV infection remodels host nucleotide metabolism in a cell type-dependent manner

To further investigate the metabolic pathways affected by PEDV infection, we performed pathway enrichment analysis based on differentially regulated metabolites in LLC-PK1 and Vero E6 cells. The results, visualized as bubble plots, revealed that PEDV infection significantly altered multiple metabolic pathways.

**Fig 2:**
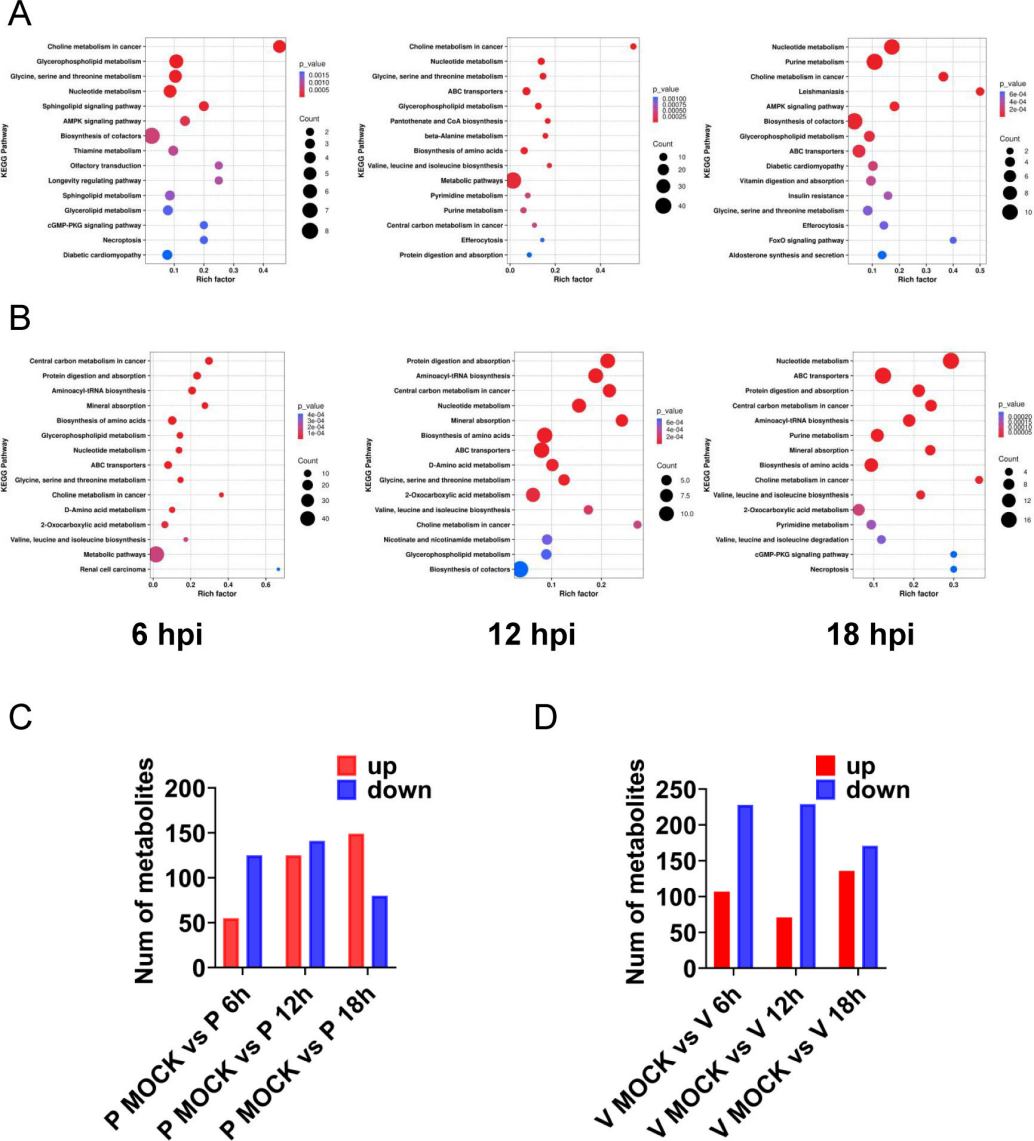
Significant differential metabolite levels and metabolic pathways in cells after PEDV infection. Bubble plots of the metabolic pathway analysis for PEDV-infected LLC-PK1 cells (**A**) and Vero E6 cells (**B**) at the indicated time points. Each bubble represents a metabolic pathway in the plot (the 15 with the highest significance, according to *P*-value, are shown). The bubble size corresponds to the number of differentially abundant metabolites enriched in each pathway. The color scale represents the *P*-value magnitude, where more intense red hues indicate smaller *P*-values and thus greater enrichment significance. Numbers of metabolites upregulated (red) and downregulated (blue) in PEDV-infected LLC-PK1(P) cells (**C**) and Vero E6(V) cells (**D**).

In LLC-PK1 cells, the most enriched pathways included choline metabolism, nucleotide metabolism, amino acid biosynthesis, purine metabolism, and glycine, serine, and threonine metabolism (Fig. 2A). In contrast, Vero E6 cells showed enrichment in nucleotide metabolism, purine metabolism, amino acid biosynthesis, and central carbon metabolism (Fig. 2B), indicating cell line-specific metabolic responses to PEDV.

Quantitative analysis revealed dynamic temporal changes in metabolite abundance. In PEDV-infected LLC-PK1 cells, compared to mock-infected controls, 55 metabolites were upregulated and 125 were downregulated at 6 hpi; 125 were upregulated and 141 were downregulated at 12 hpi; and 149 were upregulated and 80 were downregulated at 18 hpi (Fig. 2C). In Vero E6 cells, 107 metabolites were upregulated and 228 were downregulated at 6 hpi; 71 were upregulated and 229 were downregulated at 12 hpi; and 136 were upregulated and 171 were downregulated at 18 hpi (Fig. 2D). These results suggest dynamic and cell-specific metabolic reprogramming during PEDV infection.

**Fig 3:**
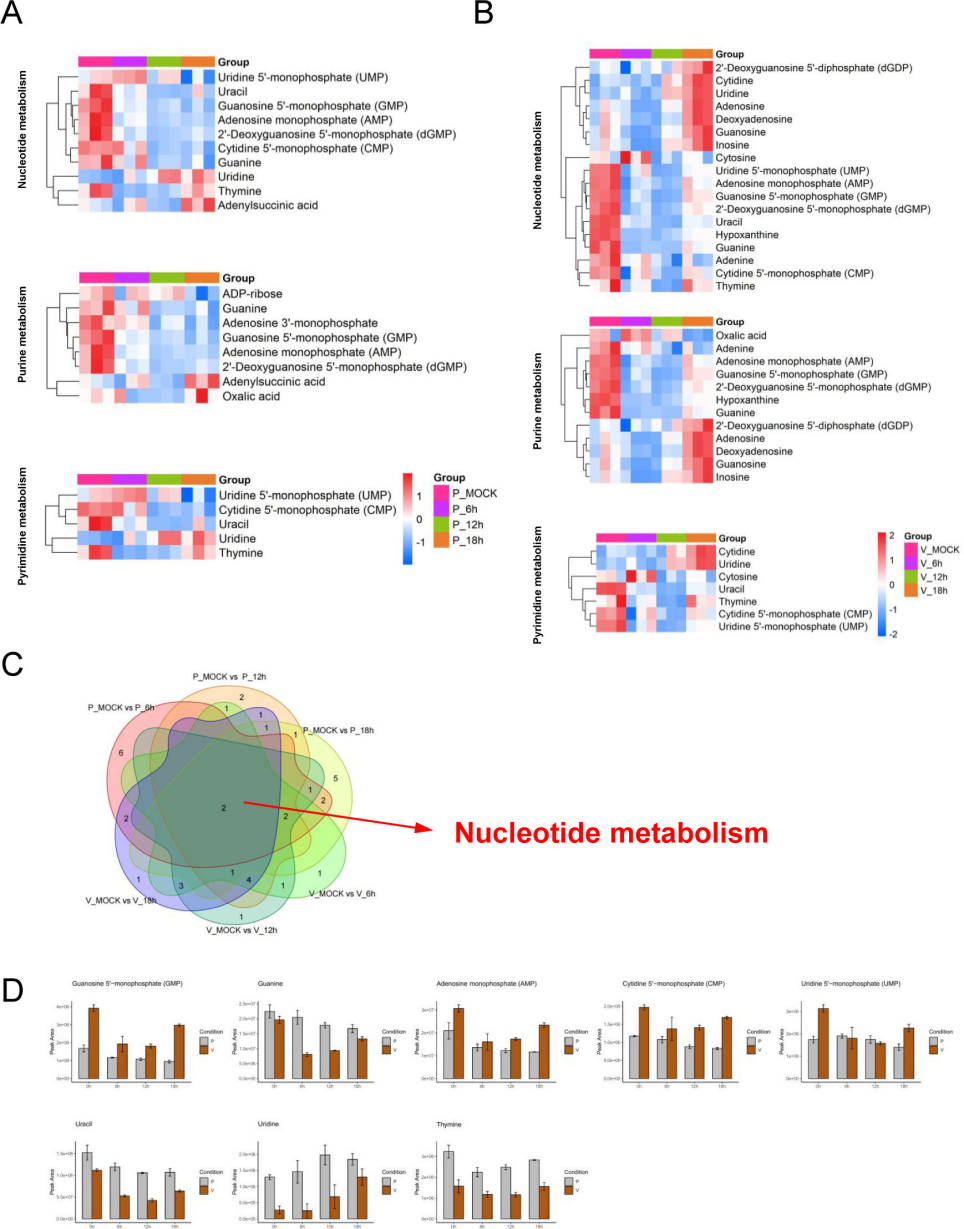
PEDV infection induces significant changes in purine and pyrimidine metabolites in LLC-PK1 and Vero E6 cells. (**A**) Heatmap of changes of the indicated metabolites between PEDV and mock-infected LLC-PK1 cells. (**B**) Heatmap of changes of the indicated metabolites between PEDV and mock-infected Vero E6 cells. The heatmap analysis was performed on software R (pheatmap). Columns correspond to individual samples, while rows represent distinct differentially abundant metabolites. The color scale denotes relative metabolite abundance levels, with red and blue indicating upregulation and downregulation, respectively. (**C**) Venn diagram showing the overlap of significantly altered metabolites between LLC-PK1 and Vero E6 cells at the indicated time points. (**D**) Bar chart of peak area of representative nucleotide monophosphates, nucleosides, and nucleobases in mock- and PEDV-infected cells in LLC-PK1(P) cells and Vero E6(V) cells.

Among the significantly altered pathways, purine and pyrimidine metabolism exhibited the most prominent changes (Fig. 3A through C; Fig. S1; Table S2). In PEDV-infected LLC-PK1 cells, the levels of GMP, AMP, CMP, UMP, guanine, and uracil were markedly decreased across all time points, while uridine and thymine were increased (Fig. 3D). In contrast, Vero E6 cells exhibited an initial decline followed by a time-dependent increase in the levels of GMP, AMP, CMP, UMP, guanine, uracil, uridine, and thymine during PEDV infection, suggesting a biphasic regulation of nucleotide metabolism (Fig. 3D). These contrasting patterns indicate that PEDV differentially regulates nucleotide metabolism in a cell-type–dependent manner, potentially reflecting differences in viral replication dynamics or host metabolic capacity.

### Inhibition of IMPDH suppresses PEDV replication

**Fig 4:**
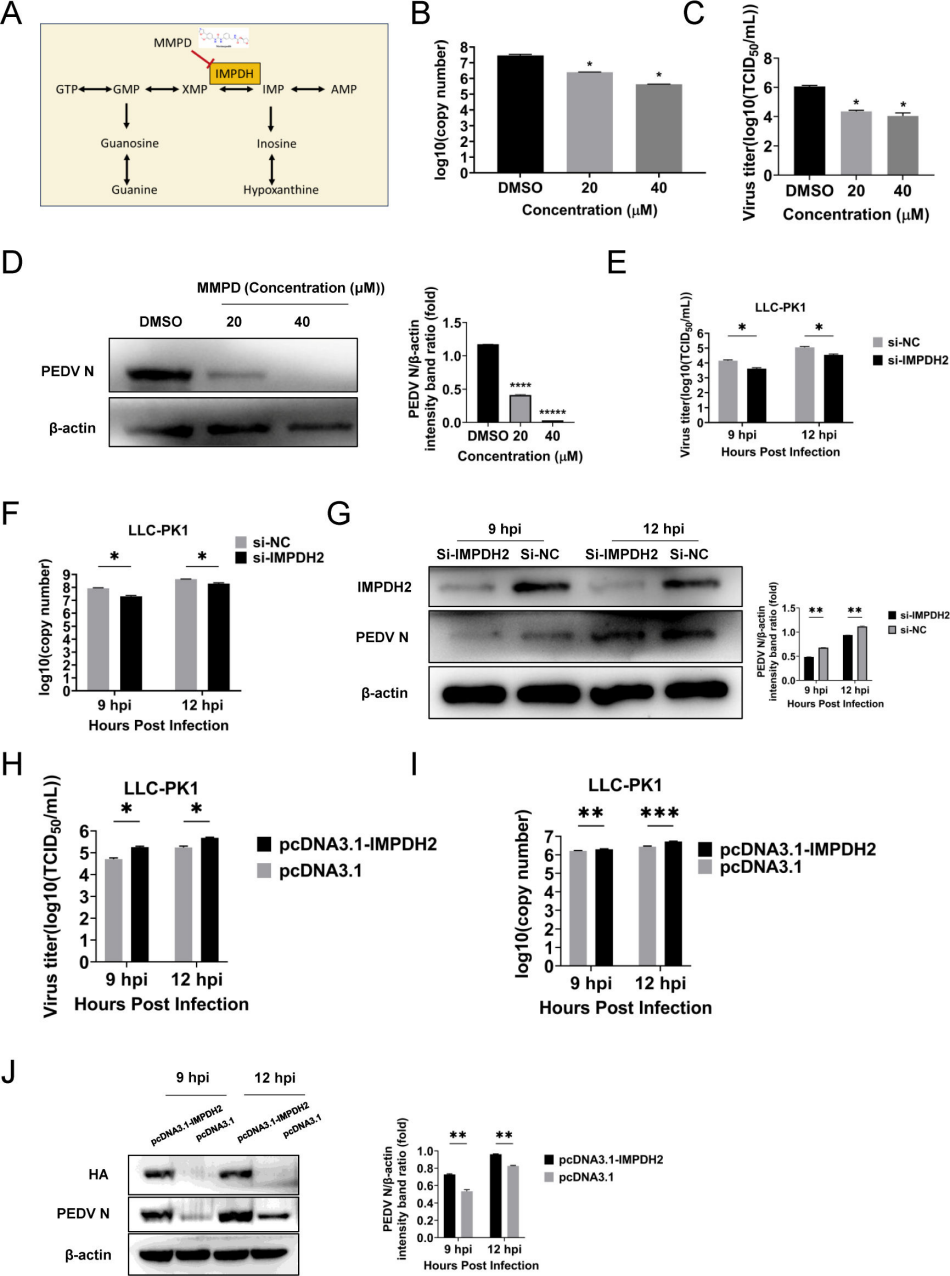
Essential role of IMPDH in the anti-PEDV activity. (**A**) Schematic representation of purine metabolism. (**B**) The PEDV genomic RNA in MMPD- or DMSO-treated LLC-PK1 cells was determined with RT-qPCR targeting the PEDV N gene. (**C**) MMPD- or DMSO-treated LLC-PK1 cells were infected with PEDV at an MOI of 0.1. The cell supernatants were collected at 12 hpi and titrated with a TCID_50_ infectivity assay. (**D**) Expression of the N protein in MMPD- or DMSO-treated LLC-PK1 cells was detected by Western blot. IMPDH siRNA was transfected into LLC-PK1 cells, and the cells were infected with PEDV at 36 h post-transfection at an MOI of 0.1. The PEDV titers were explored with TCID_50_ (**E**), RT-qPCR (**F**), and Western blot (**G**). (**H–J**) IMPDH2-overexpressing and control vector-transfected cells infected with PEDV (MOI = 0.1) at 9 and 12 hpi. PEDV titers were determined by TCID_50_ assay (**H**), PEDV genomic RNA was quantified via RT-qPCR (**I**), and N protein expression was analyzed by Western blot (**J**). Data are mean ± SD from three independent experiments. Differences were considered significant at (∗) *P* < 0.05, (∗∗) 0.001< *P* < 0.01, (∗∗∗) *P* < 0.001*.*

Based on the above results, nucleotide metabolism pathways emerged as the most strikingly enriched pathway across all six experimental groups (Fig. 3C). This observation is particularly significant given the well-established role of nucleotide biosynthesis in supporting viral replication, where IMPDH serves as the rate-limiting enzyme for guanine nucleotide production. These collective insights prompted us to evaluate the antiviral potential of merimepodib (MMPD), a selective IMPDH inhibitor, against PEDV infection in our cellular models (Fig. 4A).

Prior to antiviral assays, the cytotoxicity of MMPD was assessed in LLC-PK1, Vero E6, and IPEC-J2 cells. MMPD showed minimal cytotoxicity in all three cell lines, with 50% cytotoxic concentration (CC_50_) values of 86.42 μM, 40.13 μM, and 40.33 μM, respectively (Fig. S2). Based on these data, non-cytotoxic concentrations of MMPD at 20 and 40 μM were used for subsequent antiviral experiments.

Cells were infected with PEDV at a multiplicity of infection (MOI) of 0.1 and treated with increasing concentrations of MMPD for 12 h. RT-qPCR analysis demonstrated a dose-dependent reduction in viral RNA levels (Fig. 4B), which was further confirmed by TCID_50_ assays and Western blot (Fig. 4C and D). In LLC-PK1 cells, the half-maximal inhibitory concentration (IC_50_) of MMPD was determined to be 10.06 μM, with a selectivity index (SI) of 8.59. The IC_50_ was determined by fitting a best-fit Log(dose)-response curve. Similar inhibitory effects were observed in Vero E6 and IPEC-J2 cells (Fig. S3).

To further validate the role of IMPDH2 in PEDV replication, LLC-PK1 cells and IPEC-J2 cells were transfected with small interfering RNA (siRNA) targeting IMPDH2. Knockdown efficiency was confirmed by Western blot. At 36 h post-transfection, cells were infected with PEDV, and viral RNA levels in the supernatant were quantified at 9 and 12 h post-infection. A significant reduction in PEDV titers was observed in IMPDH2-depleted cells compared to controls (Fig. 4E; Fig. S4A), consistent with decreased genomic RNA and N protein expression (Fig. 4F and G; Fig. S4B and C). Moreover, PEDV replication was significantly enhanced following IMPDH2 overexpression in both LLC-PK1 and IPEC-J2 cells (Fig. 4H through J; Fig. S4D through F). Collectively, these findings demonstrate that IMPDH2 serves as a crucial host factor required for PEDV infection.

**Fig 5:**
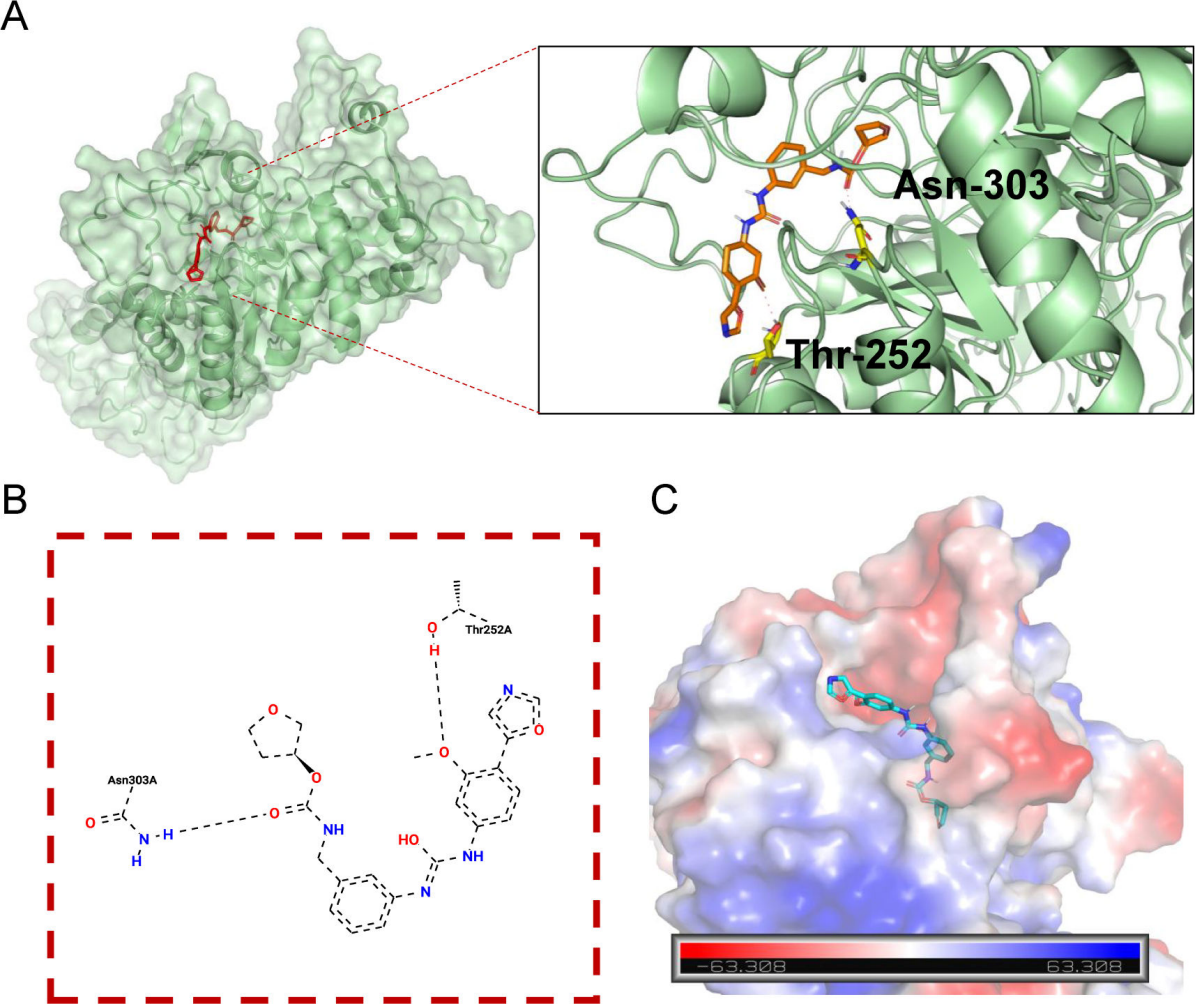
MMPD interacts with IMPDH2 *in silico*. Molecular docking experiments were performed on MMPD and IMPDH2 using AutoDock Vina (version 1.5.6) software. (**A**) Docking pose of MMPD in IMPDH. (**B**) 2D ligand interaction diagram. (**C**) Electrostatic surface potential of MMPD in IMPDH.

To investigate the molecular interaction between MMPD and IMPDH2, molecular docking was performed using AutoDock Vina. The docking model revealed that MMPD forms two hydrogen bonds with residues Thr-252 and Asn-303 of IMPDH2, suggesting a direct interaction with the enzyme’s active site (Fig. 5).

To determine whether the antiviral effect of MMPD is mediated through depletion of guanine nucleotides, a rescue experiment was conducted by supplementing MMPD-treated cells with exogenous guanosine. However, guanosine supplementation failed to restore PEDV replication in LLC-PK1 cells (Fig. S5), indicating that the antiviral effect of MMPD is not solely due to guanosine depletion, but may involve broader disruption of purine metabolism.

Collectively, these results demonstrate that both pharmacological inhibition and genetic silencing of IMPDH2 significantly impair PEDV replication *in vitro*, highlighting IMPDH2 as a promising host-directed target for anti-PEDV therapy.

### MMPD targets the post-entry stages of the PEDV life cycle

To delineate the specific stage(s) of the PEDV life cycle affected by MMPD, time-of-addition assays were performed in LLC-PK1 and IPEC-J2 cells to assess its impact on viral attachment, entry, replication, and release. Cells were treated with MMPD (30 μM) during each individual phase, and viral replication was evaluated by quantifying PEDV genomic RNA in culture supernatants using RT-qPCR, along with determination of infectious titers by TCID_50_ assay.

**Fig 6:**
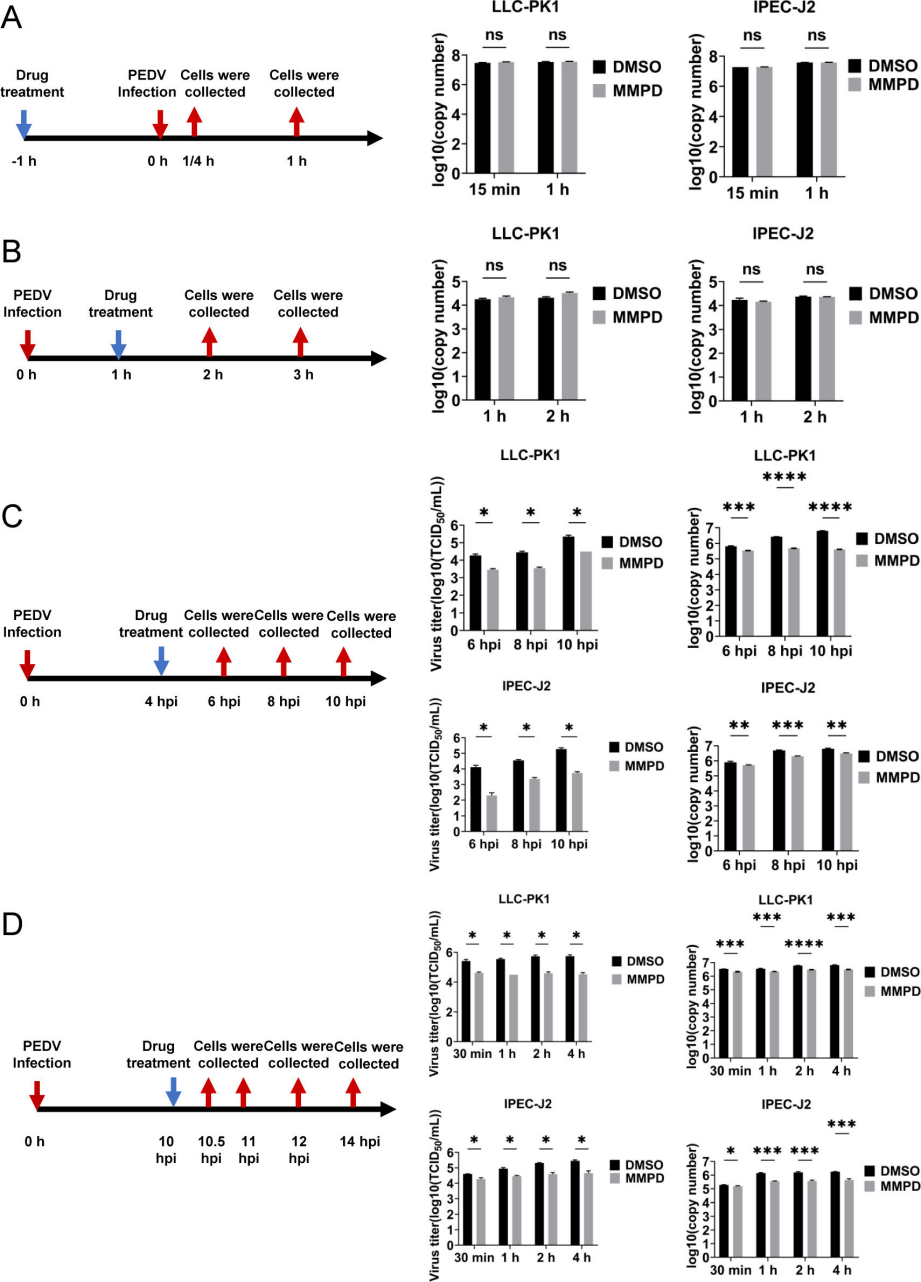
The antiviral effect of MMPD treatment on different infection steps of PEDV. LLC-PK1 cells and IPEC-J2 cells were infected with PEDV *SHpd/2012* and treated with MMPD at specific time points corresponding to different stages of the viral life cycle: attachment (**A**), internalization (**B**), replication (**C**), and release (**D**). At indicated time points, samples were collected to determine progeny virus titers using the TCID_50_ assay and to quantify PEDV genomic RNA levels by RT-qPCR. Data are mean ± SD from three independent experiments. Differences were considered significant at (∗) *P* < 0.05, (∗∗) 0.001 < *P* < 0.01, (∗∗∗) *P* < 0.001, (∗∗∗∗) *P* < 0.0001, ns, not significant*.*

No significant differences in viral RNA levels were observed between the MMPD-treated and DMSO control groups when MMPD was applied during the attachment or internalization phases (Fig. 6A and B). In contrast, MMPD treatment during the replication and release phases resulted in a marked reduction in both viral RNA levels and infectious titers (Fig. 6C and D). Consistently, Western blot analysis showed decreased expression of the viral nucleocapsid (N) protein in cells treated with MMPD during the post-entry phases (Fig. S6).

Taken together, these findings indicate that MMPD exerts its antiviral effect by interfering with the replication and release stages of the PEDV life cycle, with no significant impact on viral attachment or internalization.

### *In vitro* efficacy of MMPD against multiple PEDV genotypes

To evaluate the broader antiviral potential of MMPD, its efficacy was assessed against different genotypes of PEDV in three cell lines. LLC-PK1 cells were first infected with either the genotype 1 (G1) strain HLJ or the S-INDEL strain AH2018 for 1 h, followed by treatment with 20 or 40 µM MMPD for 12 h.

**Fig 7:**
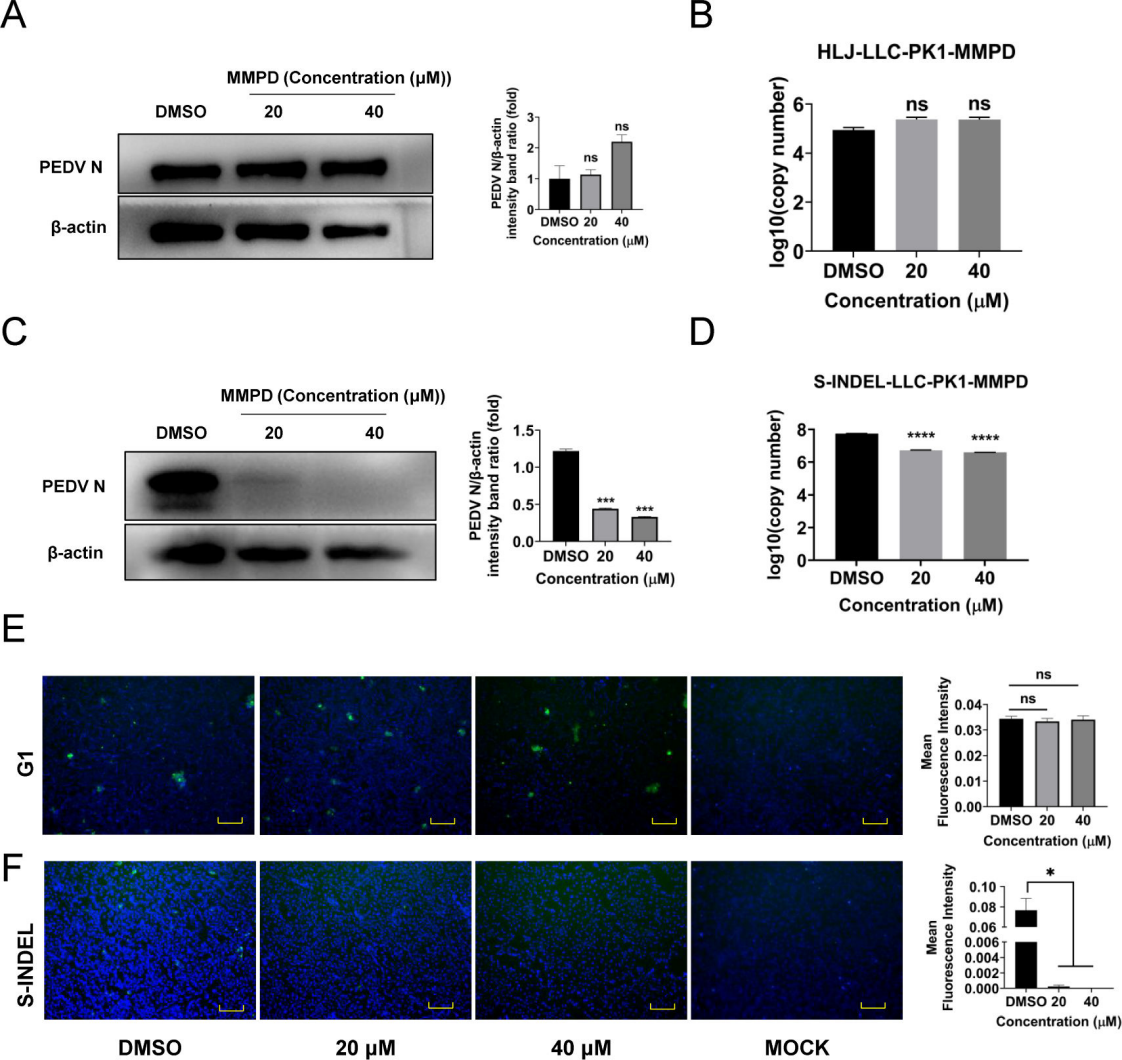
Antiviral activity of CFZ against different genotypes of PEDV strains at an MOI of 0.1 in LLC-PK1 cells. (**A**) Effect of MMPD on N protein expression of PEDV G1 strains by Western blot. (**B**) Effect of MMPD on viral RNA synthesis of PEDV G1 strains. (**C**) Effect of MMPD on N protein expression of PEDV S-INDEL strains by Western blot. (**D**) Effect of MMPD on viral RNA synthesis of PEDV S-INDEL strains. (**E**) PEDV N protein expression of G1 strains was determined by IFA. (**F**) PEDV N protein expression of S-INDEL strains was determined by IFA. Scale bars = 100 µm. Data are mean ± SD from three independent experiments. Differences were considered significant at (∗) *P* < 0.05, (∗∗) 0.001 < *P* < 0.01, (∗∗∗) *P* < 0.001, (∗∗∗∗) *P* < 0.0001, ns, not significant*.*

As shown in Fig. 7A, B and E, MMPD treatment did not significantly reduce viral replication of the HLJ strain in LLC-PK1 cells. In contrast, MMPD markedly suppressed replication of the AH2018 strain in the same cell line, as evidenced by reduced viral RNA levels and N protein expression (Fig. 7C, D and F).

To further confirm these findings, IPEC-J2 and Vero E6 cells were similarly treated and infected with both PEDV strains. Western blot analysis revealed a significant reduction in N protein levels in MMPD-treated cells infected with either strain. Consistent results were observed in RT-qPCR and immunofluorescence assays (IFA), supporting the broad antiviral effect of MMPD in both IPEC-J2 and Vero E6 cells (Fig. S7).

Collectively, these data demonstrate that MMPD exhibits genotype-dependent antiviral activity against PEDV and retains efficacy in multiple porcine and non-porcine cell models. These findings suggest that IMPDH inhibition by MMPD may serve as a promising host-targeted strategy against emerging and genetically diverse PEDV strains.

### IMPDH inhibition disrupts nucleotide metabolism during PEDV infection

To investigate how IMPDH influences PEDV replication through host metabolic regulation, untargeted metabolomic profiling was conducted using UHPLC-MS/MS. LLC-PK1 cells were divided into three groups: a DMSO-treated control (MOCK), cells transfected with siRNA targeting IMPDH2, and cells treated with merimepodib (MMPD). All groups were subsequently infected with PEDV at a MOI of 0.1, and intracellular metabolites were analyzed post-infection.

**Fig 8:**
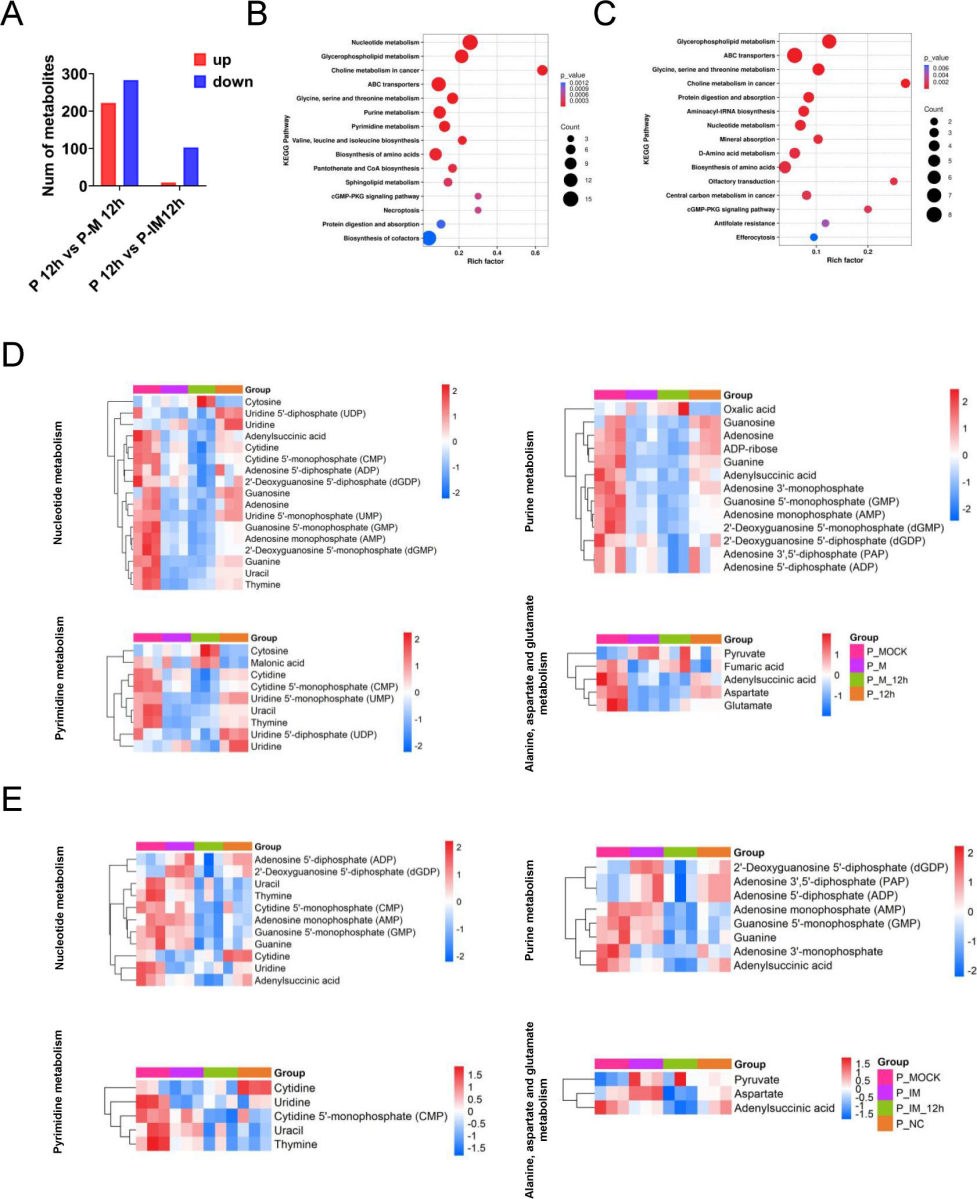
Impact of IMPDH2 inhibitors on host nucleotide metabolism. (**A**) Numbers of metabolites upregulated (red) and downregulated (blue) in PEDV-infected LLC-PK1 cells. (**B**) Bubble plots of the metabolic pathway analysis for MMPD-treated PEDV-infected cells. (**C**) Bubble plots of the metabolic pathway analysis for siRNA-mediated IMPDH2 knockdown cells. Each bubble represents a metabolic pathway in the plot (the 15 with the highest significance, according to *P-*value, are shown). The bubble size corresponds to the number of differentially abundant metabolites enriched in each pathway. The color scale represents the *P*-value magnitude, where more intense red hues indicate smaller *P*-values and thus greater enrichment significance. Heatmap of changes of the indicated metabolites for MMPD-treated PEDV-infected LLC-PK1 cells (**D**) and siRNA-mediated IMPDH2 knockdown cells (**E**). The heatmap analysis was performed on software R (pheatmap). Columns correspond to individual samples, while rows represent distinct differentially abundant metabolites. The color scale denotes relative metabolite abundance levels, with red and blue indicating upregulation and downregulation, respectively.

Metabolomic analysis revealed significant differences in metabolic profiles among the three groups. MMPD treatment altered a total of 505 metabolites (222 upregulated, 283 downregulated) compared with PEDV-infected controls, whereas IMPDH2 knockdown led to differential expression of 112 metabolites (nine upregulated, 103 downregulated) (Fig. 8A).

Pathway enrichment analysis using the KEGG database demonstrated that MMPD-induced metabolic changes were primarily enriched in nucleotide metabolism, glycerophospholipid metabolism, choline metabolism in cancer, and ABC transporter pathways (Fig. 8B). Similarly, the IMPDH2 knockdown group showed enrichment in glycerophospholipid metabolism, ABC transporters, and nucleotide metabolism compared to the infected control (Fig. 8C).

Further comparison of the metabolite profiles revealed that both MMPD treatment and IMPDH2 silencing led to a marked suppression of purine metabolism, as evidenced by a selective reduction in key metabolites within the nucleotide metabolism pathway (Fig. 8D and E).

Together, these data indicate that inhibition of IMPDH activity—either pharmacologically or genetically—disrupts host purine metabolism, thereby impairing PEDV replication. These findings highlight the critical role of nucleotide biosynthesis in PEDV infection and further support IMPDH as a viable metabolic target for antiviral intervention.

## DISCUSSION

Viruses have evolved sophisticated strategies to hijack host cell metabolism, ensuring a sufficient supply of energy and biosynthetic precursors to support their replication (25, 26). With recent advances in metabolomics technologies, our ability to investigate virus-induced metabolic reprogramming has substantially improved (27). In this study, we employed untargeted UHPLC-MS/MS–based metabolomics to characterize the global metabolic changes induced by PEDV infection. Our results revealed significant alterations in host cellular metabolism, with purine and pyrimidine metabolism being among the most profoundly affected pathways. These findings suggest that PEDV manipulates host nucleotide biosynthesis to meet the increased demand for RNA precursors required for efficient viral replication.

We profiled metabolic alterations in two PEDV-susceptible cell lines, LLC-PK1 and Vero E6, and observed distinct but overlapping metabolic remodeling in response to infection. Using OPLS-DA and TIC chromatograms, we confirmed the reliability of the metabolic data sets. KEGG enrichment analysis of differentially abundant metabolites further revealed key pathways modulated by PEDV, including nucleotide metabolism, amino acid biosynthesis, choline metabolism, and purine metabolism.

Notably, the metabolic responses to PEDV infection were cell-type dependent. In LLC-PK1 cells, the levels of several nucleotide-related metabolites, such as GMP, AMP, and CMP, were significantly downregulated over time, while uridine and thymine levels increased. In contrast, Vero E6 cells exhibited a biphasic pattern: a rapid early depletion, followed by a time-dependent increase in the same nucleotide metabolites. These observations indicate that PEDV induces dynamic and divergent metabolic rewiring in different host cell types, possibly reflecting differences in replication kinetics or metabolic plasticity.

Among the altered pathways, *de novo* nucleotide biosynthesis emerged as a critical metabolic node for PEDV replication. In particular, we focused on IMPDH, the rate-limiting enzyme in guanine nucleotide biosynthesis. Pharmacological inhibition of IMPDH with merimepodib (MMPD), as well as siRNA-mediated knockdown of IMPDH2, resulted in a significant reduction in PEDV replication in all tested cell lines, including LLC-PK1, Vero E6, and IPEC-J2. Metabolomic profiling of MMPD-treated or IMPDH2-depleted cells confirmed a pronounced reduction in purine metabolites, providing functional evidence that IMPDH activity is essential for maintaining the nucleotide pool required for PEDV RNA synthesis. Interestingly, it seems that the knockdown exhibited a delayed reduction in metabolites compared to the inhibitor-treated infection (Fig. 8A). We propose that this difference stems from their distinct mechanisms of action—inhibitors typically target enzyme activity directly and can rapidly block metabolic pathways, whereas gene knockdown reduces enzyme levels, requiring waiting for the degradation of existing proteins (28, 29). More importantly, inhibitors, as candidate drugs, offer rapid response which may support their therapeutic advantage, whereas the delayed action of knockdown suggests that gene therapy necessitates earlier intervention.

In addition to purine metabolism, IMPDH inhibition also altered other metabolic pathways, such as glycerophospholipid metabolism and ABC transporter activity, suggesting broader metabolic dysregulation that may impair viral assembly or egress. Molecular docking analysis revealed that MMPD forms stable interactions with IMPDH2 via hydrogen bonds at residues Thr-252 and Asn-303, offering mechanistic insight into its inhibitory effect. Importantly, guanosine supplementation failed to rescue PEDV replication in MMPD-treated cells, suggesting that PEDV imposes a high nucleotide demand that cannot be readily compensated by exogenous guanosine, or that additional regulatory checkpoints may exist beyond simple metabolite availability.

These findings are consistent with previous reports showing that IMPDH plays a key role in the replication of other RNA viruses, including Zika virus, hepatitis C virus, and Mpox virus (20, 21), highlighting its potential as a broad-spectrum antiviral target. Our results further demonstrate that MMPD exerts a dose-dependent inhibitory effect on PEDV replication, as confirmed by reductions in viral RNA (RT-qPCR), viral protein levels (Western blot), and infectious titers (TCID_50_). Time-of-addition experiments indicated that MMPD specifically interferes with the replication and release phases of the viral life cycle, rather than with viral attachment or entry.

Interestingly, the antiviral activity of MMPD varied among PEDV strains and host cell types. In LLC-PK1 cells, MMPD was more effective against the S-INDEL strain AH2018 than the classical G1 strain HLJ. However, robust antiviral effects were observed for both strains in IPEC-J2 and Vero E6 cells. These findings suggest that host cell context and viral genotype may both influence the efficacy of metabolic inhibitors and underscore the need for further evaluation in physiologically relevant *in vivo* models.

In summary, our study provides compelling evidence that PEDV extensively reprograms host metabolism to facilitate its replication, with nucleotide biosynthesis—and specifically IMPDH activity—playing a central role. Targeting this pathway using IMPDH inhibitors, such as MMPD, significantly impairs PEDV replication *in vitro*, offering a promising antiviral strategy. Given that MMPD has already undergone clinical evaluation in humans, its repurposing for veterinary antiviral use may represent a rapid translational path to combat emerging PEDV outbreaks. Further studies are warranted to explore the therapeutic potential of IMPDH inhibition *in vivo* and to assess its efficacy across a broader range of porcine coronaviruses.

## Data Availability

Raw metabolomics mass spectrometry data were deposited in MetaboLights (https://www.ebi.ac.uk/metabolights/, MTBLS13153).

## SUPPLEMENTAL MATERIAL

Figure S1 jvi.01736-25-s0001.tif PEDV infection alters host metabolites in LLC-PK1 cells and Vero E6 cells.

Figure S2 jvi.01736-25-s0002.tif Evaluation of MMPD cytotoxicity.

Figure S3 jvi.01736-25-s0003.tif MMPD inhibits PEDV infection in IPEC-J2 and Vero E6 cells.

Figure S4 jvi.01736-25-s0004.tif IMPDH2 knockdown inhibits the replication stage of PEDV in IPEC-J2 cells.

Figure S5 jvi.01736-25-s0005.tif Effects of graded guanosine doses on PEDV replication in guanine-pretreated LLC-PK1 cells.

Figure S6 jvi.01736-25-s0006.tif Antiviral effect of MMPD on replication and release steps of PEDV.

Figure S7 jvi.01736-25-s0007.tif Antiviral activity of MMPD against different genotypes of PEDV strains at an MOI of 0.1 in IPEC-J2 and Vero E6 cells.

Supplemental legends and Table S1 jvi.01736-25-s0008.docx Legends for Fig. S1 to S7; Table S1, primers.

Table S2 jvi.01736-25-s0009.xlsx Differentially expressed metabolites.

ASM does not own the copyrights to Supplemental Material that may be linked to, or accessed through, an article. The authors have granted ASM a non-exclusive, world-wide license to publish the Supplemental Material files. Please contact the corresponding author directly for reuse.
