# Supplementary figures and images for: Porcine epidemic diarrhea virus manipulates IMPDH-dependent nucleotide biosynthesis to facilitate replication
Source: J Virol. 2026 Jan 9;100(2):e01736-25. doi: 10.1128/jvi.01736-25 (PMC12911865; doi:10.1128/jvi.01736-25)

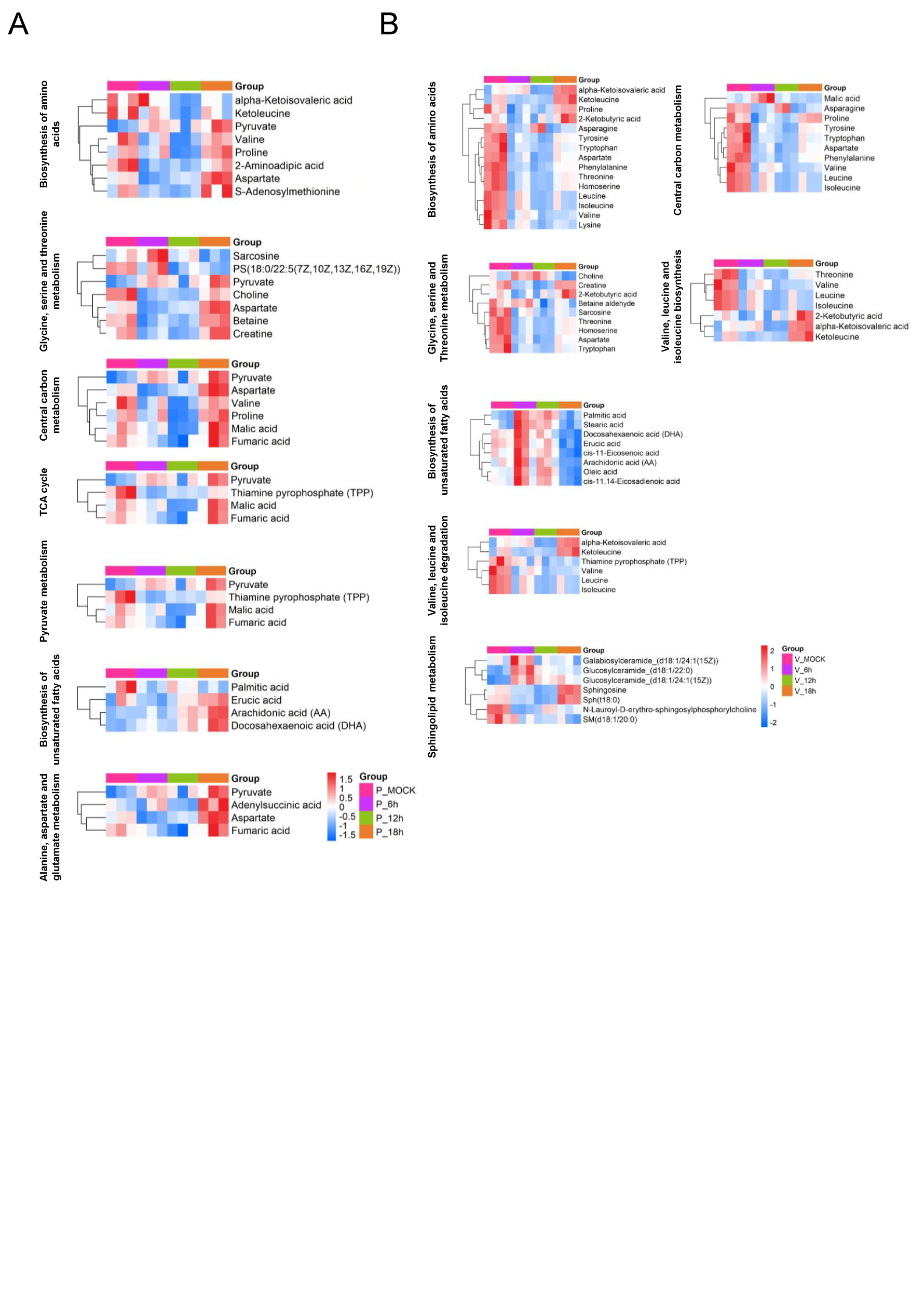

Supplement: Figure S1 — PEDV infection alters host metabolites in LLC-PK1 cells and Vero E6 cells. [file jvi.01736-25-s0001.tif]

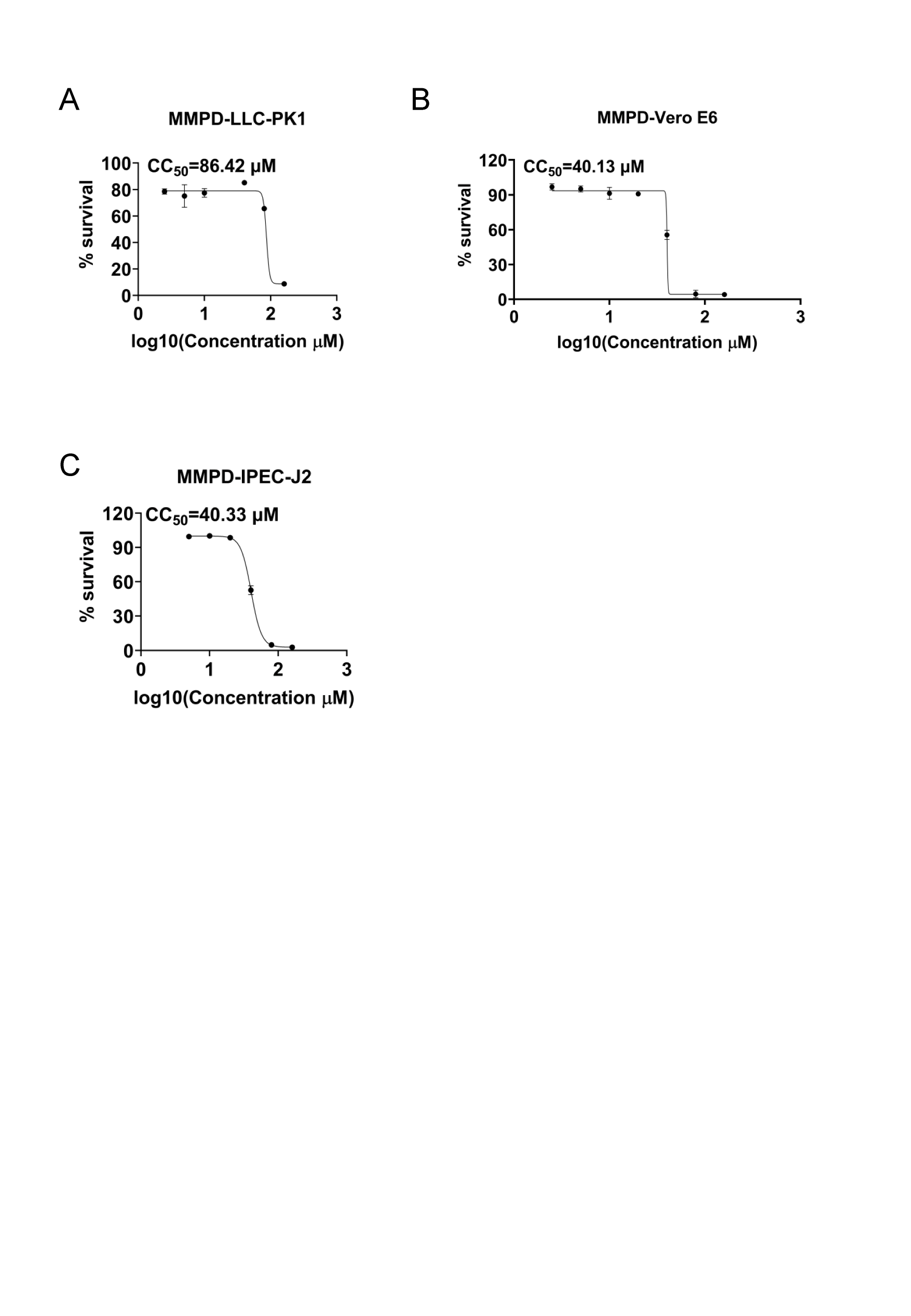

Supplement: Figure S2 — Evaluation of MMPD cytotoxicity. [file jvi.01736-25-s0002.tif]

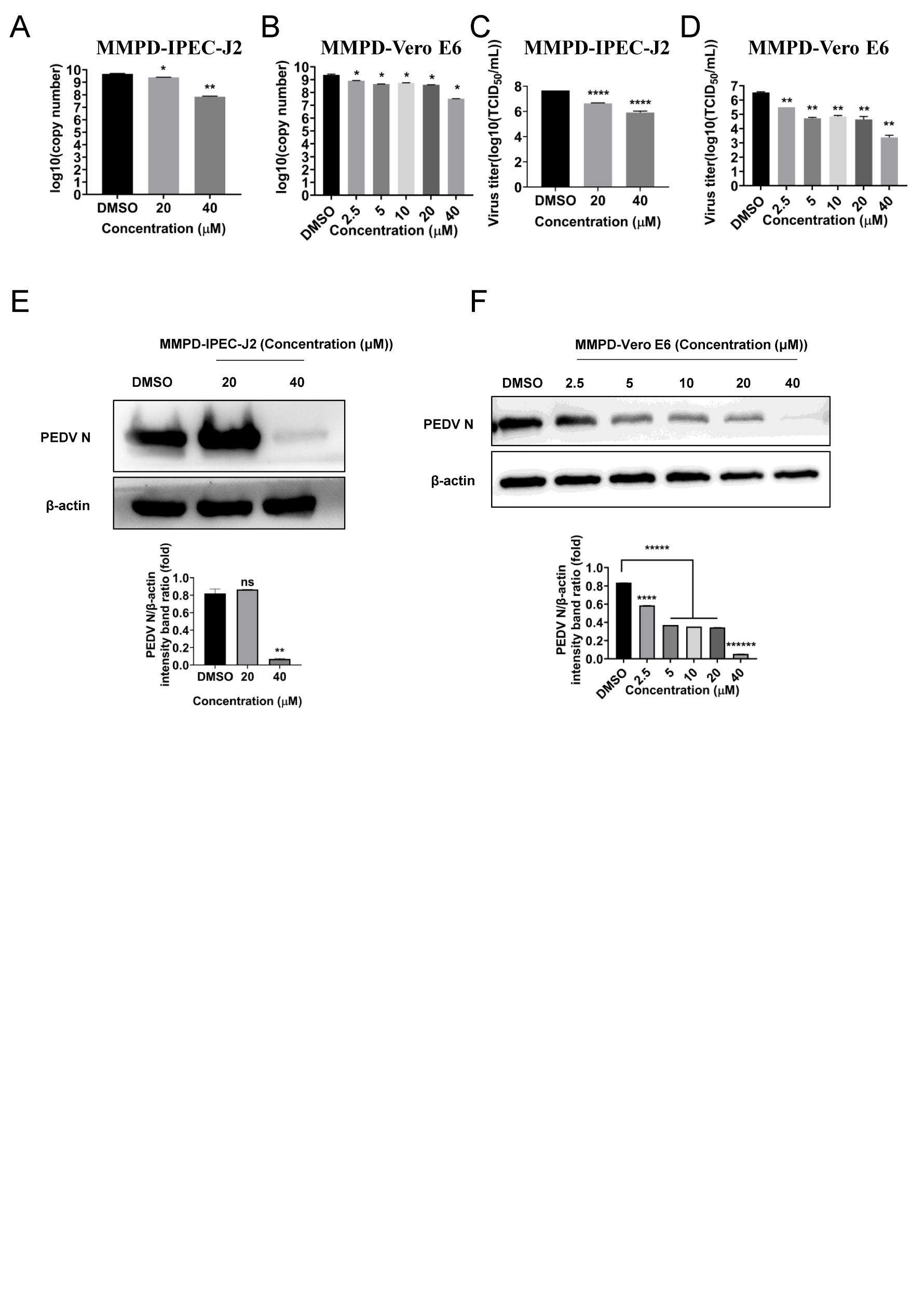

Supplement: Figure S3 — MMPD inhibits PEDV infection in IPEC-J2 and Vero E6 cells. [file jvi.01736-25-s0003.tif]

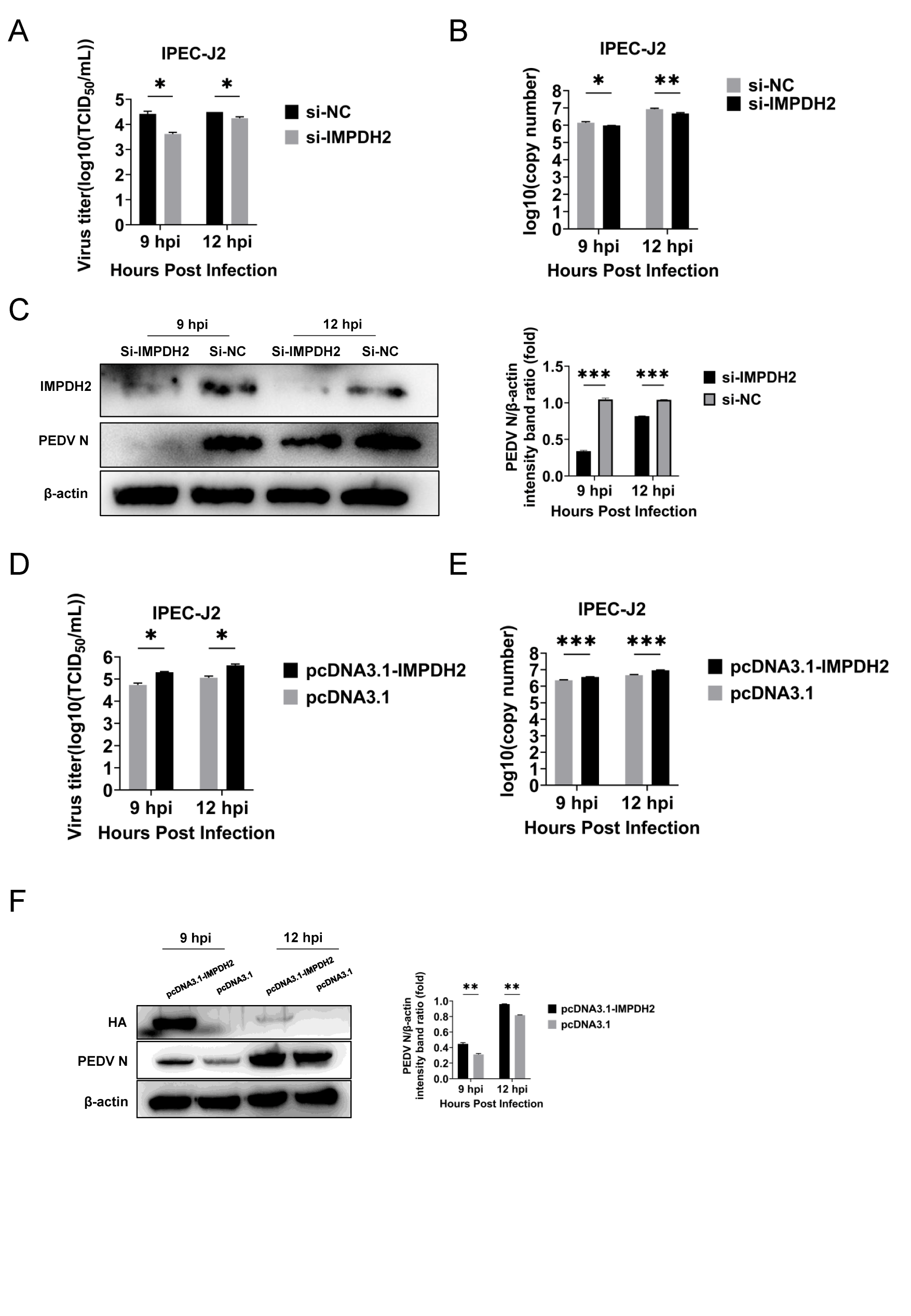

Supplement: Figure S4 — IMPDH2 knockdown inhibits the replication stage of PEDV in IPEC-J2 cells. [file jvi.01736-25-s0004.tif]

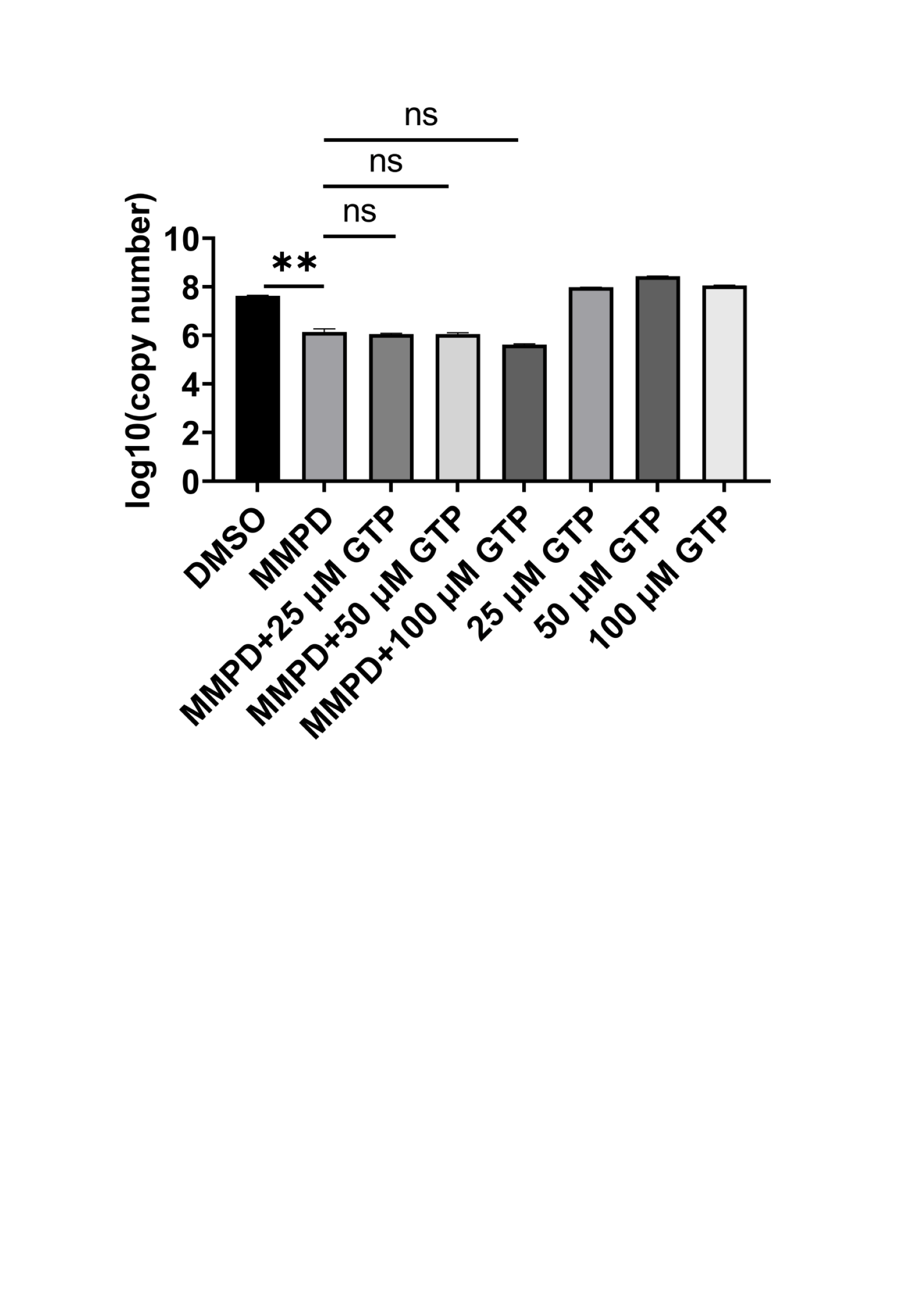

Supplement: Figure S5 — Effects of graded guanosine doses on PEDV replication in guanine-pretreated LLC-PK1 cells. [file jvi.01736-25-s0005.tif]

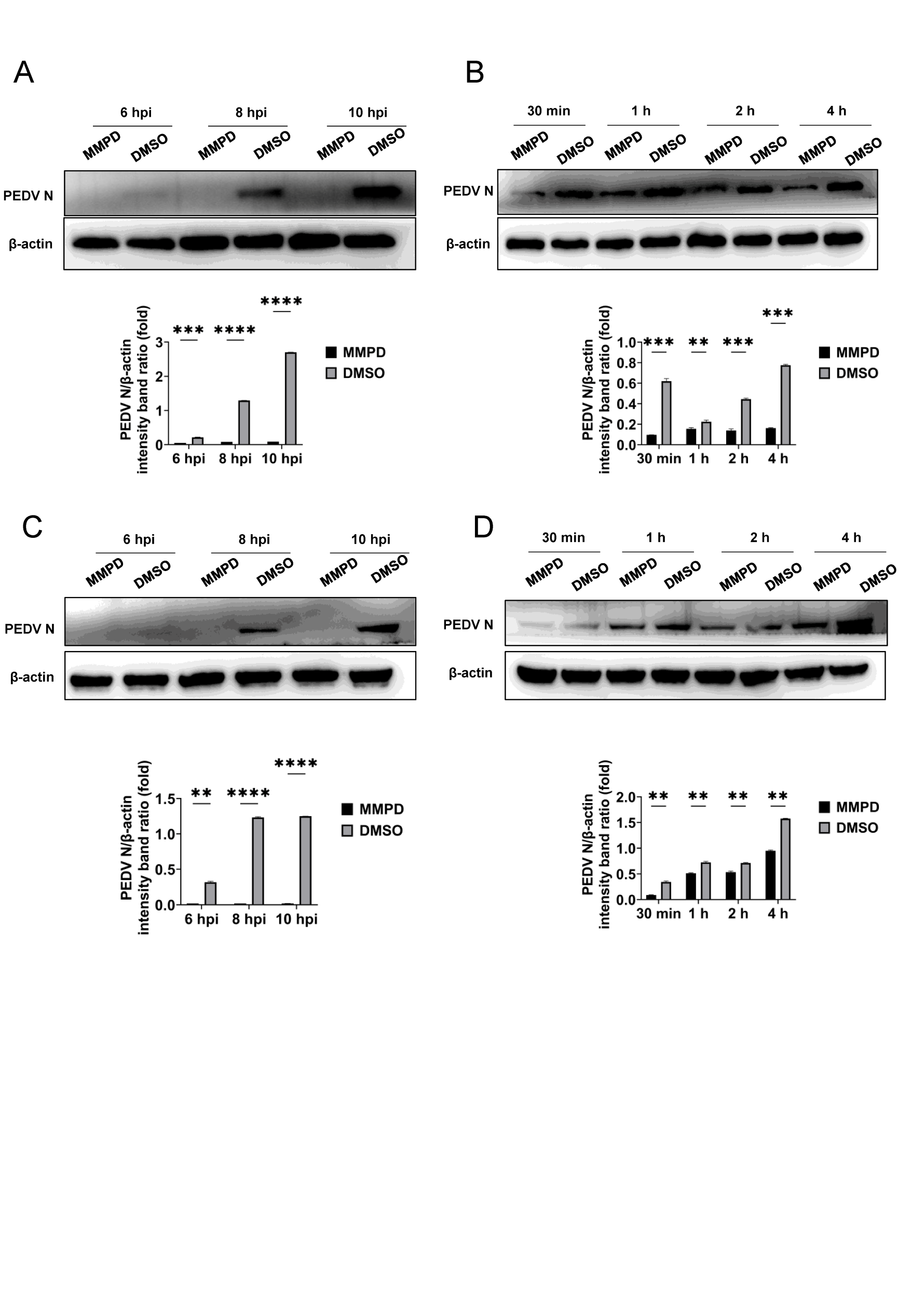

Supplement: Figure S6 — Antiviral effect of MMPD on replication and release steps of PEDV. [file jvi.01736-25-s0006.tif]

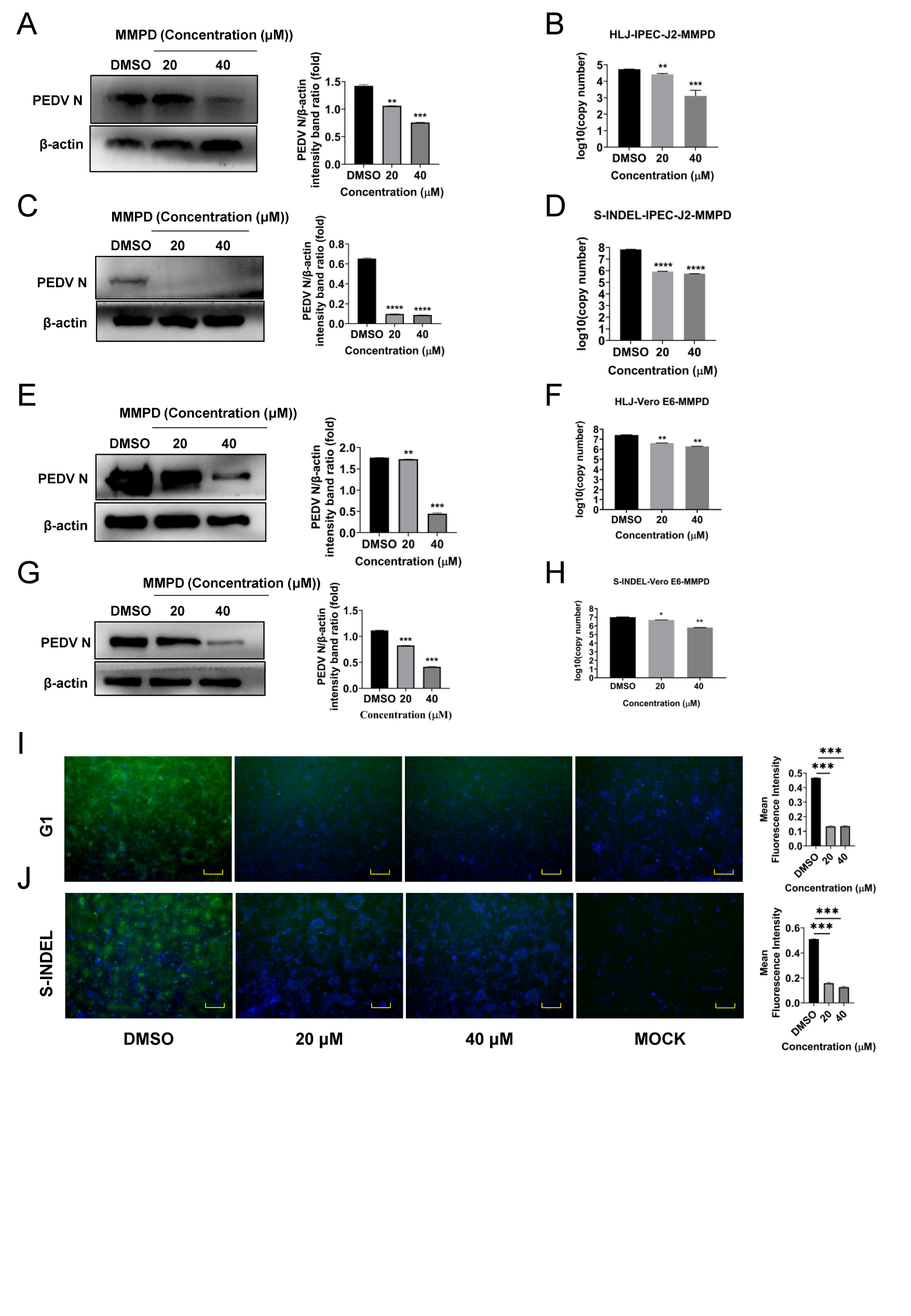

Supplement: Figure S7 — Antiviral activity of MMPD against different genotypes of PEDV strains at an MOI of 0.1 in IPEC-J2 and Vero E6 cells. [file jvi.01736-25-s0007.tif]
