# Supplementary material for: Porcine epidemic diarrhea virus manipulates IMPDH-dependent nucleotide biosynthesis to facilitate replication
Source: J Virol. 2026 Jan 9;100(2):e01736-25. doi: 10.1128/jvi.01736-25 (PMC12911865; doi:10.1128/jvi.01736-25)
Supplement: Supplemental legends and Table S1 — Legends for Fig. S1 to S7; Table S1, primers. [file jvi.01736-25-s0008.docx]

***Journal of Virology***

**Supplementary Data**

**Porcine epidemic diarrhea virus manipulates IMPDH-dependent nucleotide biosynthesis to facilitate replication**

**Shuting Zhou****^a^, Houde Zhao^a^, Junrui Zhu^b^, Yanjun Zhou^b*^, Zhibiao Yang^ac*^, Zhe Wang^ac*^**

^a^Shanghai Collaborative Innovation Center of Agri-Seeds / School of Agriculture and Biology, Shanghai Jiao Tong University, Shanghai 200240, China.

^b^Shanghai Veterinary Research Institute, Chinese Academy of Agricultural Sciences, Shanghai 200241, China.

^c^Shanghai Key Laboratory of Veterinary Biotechnology, School of Agriculture and Biology, Shanghai Jiao Tong University, Shanghai 200240, China.

E-mail addresses: [yjzhou@shvri.ac.cn](mailto:yjzhou@shvri.ac.cn) (Y.-J. Zhou), zbyang@sjtu.edu.cn (Z.-B. Yang), wangz@sjtu.edu.cn (Z. Wang).

**ORCID: 0000-0002-2524-0831 (****Y.-J. Zhou), 0000-0003-0564-1977 (Z.-B. Yang), 0000-0002-7235-3046 (Z. Wang)**

**Supplementary Figure S1**. PEDV infection alter host metabolites in LLC-PK1 cells and Vero E6 cells. Heatmap of changes of the indicated metabolites between PEDV and mock-infected cells in LLC-PK1(A) and Vero E6(B). The heatmap analysis was performed on software R(pheatmap). Columns correspond to individual samples, while rows represent distinct differentially abundant metabolites. The color scale denotes relative metabolite abundance levels, with red and blue indicating upregulation and downregulation, respectively.

**Supplementary Figure S2**. Evaluation of MMPD’s cytotoxicity. Dose-dependent curves showed viability of LLC-PK1 cells(A), Vero E6 cells (B), and IPEC-J2 cells (C) with serial dilution concentrations of MMPD. DMSO-treated cells were used as a control, with cell viability normalized to 100%.

**Supplementary Figure S3**. MMPD inhibits PEDV infection in IPEC-J2 and Vero E6 cells. The PEDV genomic RNA in MMPD or DMSO treated IPEC-J2 cells (A) and Vero E6 cells (B) were determined with RT-qPCR targeting the PEDV N gene. MMPD or DMSO treated cells were infected with PEDV at an MOI of 0.1. The IPEC-J2 cell (C) and Vero E6 (D) supernatants were collected at 12 hpi and titrated with a TCID_50_ infectivity assay. Expression of the N protein in MMPD or DMSO treated IPEC-J2 cells (E) and Vero E6 cells (F) was detected by Western blot. Data are mean ± SD from three independent experiments. Differences were considered significant at *(∗) P < 0.05, (∗∗) 0.001<P < 0.01, (∗∗∗) P < 0.001*.

**Supplementary Figure S4.** IMPDH2 Knockdown inhibits the replication stage of PEDV in IPEC-J2 cells. IMPDH siRNA transfected into IPEC-J2 cells and infected with PEDV at 36 h post-transfection at the MOI of 0.1. The PEDV titers were explored with TCID_50_ (A), RT-qPCR (B), and Western blot (C). (D-F) IMPDH2-overexpressing and control vector-transfected cells infected with PEDV (MOI = 0.1) at 9 and 12 hpi. PEDV titers were determined by TCID_50_ assay (D), PEDV genomic RNA were quantified via RT-qPCR (E), and N protein expression was analyzed by Western blot (F). Data are mean ± SD from three independent experiments. Differences were considered significant at *(∗) P < 0.05, (∗∗) 0.001<P < 0.01, (∗∗∗) P < 0.001*.

**Supplementary Figure S5.** Effects of graded guanosine doses on PEDV replication in guanine-pretreated LLC-PK1 cells. After 6-hour guanine pretreatment, LLC-PK1 cells infected with 0.1 MOI PEDV *SHpd/2012* were treated with MMPD plus graded guanosine doses (25, 50, and 100 μM). PEDV RNA was measured by RT-qPCR at 12 hpi. Data are mean ± SD from three independent experiments. Differences were considered significant at *(∗) P < 0.05, (∗∗) 0.001<P < 0.01, (∗∗∗) P < 0.001*.

**Supplementary Figure S6.** The antiviral effect of MMPD on replication and release steps of PEDV. N protein expression in MMPD- or DMSO-treated LLC-PK1 cells (A-B) and IPEC-J2 cells (C-D) was examined by Western blot at replication (A, C) and release (B, D) phases.

**Supplementary Figure S7.** Antiviral activity of MMPD against different genotypes of PEDV strains at an MOI of 0.1 in IPEC-J2 and Vero E6 cells. Effect of MMPD on N protein expression of PEDV G1 strains by Western blot in IPEC-J2 (A) and Vero E6 cells (E). Effect of MMPD on N protein expression of PEDV S-INDEL strains by Western blot in IPEC-J2 (C) and Vero E6 cells (G). Effect of MMPD on viral RNA synthesis of PEDV G1 strains in IPEC-J2 (B) and Vero E6 cells (F). Effect of MMPD on viral RNA synthesis of PEDV S-INDEL strains in IPEC-J2 (D) and Vero E6 cells (H). (I) PEDV N protein expression of G1 strains in Vero E6 was determined by IFA. (J) PEDV N protein expression of S-INDEL strains in Vero E6 was determined by IFA. Scale bars=100 μm. Data are mean ± SD from three independent experiments. Differences were considered significant at *(∗) P < 0.05, (∗∗) 0.001<P < 0.01, (∗∗∗) P < 0.001*.

**Table S1.** List of primers used in this study.

| Primer Name | Sequence (5’-3’) |
| --- | --- |
| qPCR-PEDV-N-F | CGCAAAGACTGAACCCACTAAC |
| qPCR-PEDV-N-R | TTGCCTCTGTTGTTACTTGGAGAT |
| qPCR-GAPDH-monkey-F | ACAACAGCCTCAAGATCGTCAGC |
| qPCR-GAPDH-monkey-R | GTGGCAGTGATGGCGTGGAC |
| qPCR-GAPDH-pig-F | GGAAGCTGTGGCGTGATGGC |
| qPCR-GAPDH-pig-R | TTCTCCAGGCGGCAGGTCAG |
| si-IMPDH-sense | AUGGCGGACUACCUGAUCATT |
| si-IMPDH-antisense | UGAUCAGGUAGUCCGCCAUTT |
